# MOTS-c Levels and Sarcopenia Risk in Chronic Peritoneal Dialysis Patients: A Pilot Study

**DOI:** 10.3390/medicina61020322

**Published:** 2025-02-12

**Authors:** Mariateresa Zicarelli, Marta Greco, Stefanos Roumeliotis, Maria Elisa Lo Vasco, Francesco Dragone, Christodoula Kourtidou, Ioannis Alekos, Roberta Misiti, Daniela Patrizia Foti, Giuseppe Coppolino, Vassilios Liakopoulos, Evangelia Dounousi, Davide Bolignano

**Affiliations:** 1 Department of Health Sciences, Magna-Graecia University, 88100 Catanzaro, Italy; 2 Clinical Pathology Lab, Magna-Graecia University Hospital, 88100 Catanzaro, Italy; 3 2nd Department of Nephrology, AHEPA University Hospital Medical School, Aristotle University of Thessaloniki, 541 24 Thessaloniki, Greece; 4 School of Medicine, Magna-Graecia University, 88100 Catanzaro, Italy; 5 Department of Nephrology, School of Medicine, University of Ioannina, 451 10 Ioannina, Greece; 6 Department of Experimental and Clinical Medicine, Magna-Graecia University, 88100 Catanzaro, Italy; 7 Department of Medical and Surgical Sciences, Magna-Graecia University, 88100 Catanzaro, Italy

**Keywords:** sarcopenia, ESKD, peritoneal dialysis, MOTS-c, SARC-F

## Abstract

*Background and Objectives:* Sarcopenia is exceedingly frequent in end-stage kidney disease (ESKD) patients on dialysis, including those undergoing peritoneal dialysis (PD), and is of multifactorial origin. MOTS-c is a mitochondrial-derived peptide that promotes muscle growth whose levels are unbalanced in ESKD. In this study, we evaluated MOTS-c balance and its relationship with sarcopenia risk in an ESKD-PD cohort. *Materials and Methods:* MOTS-c was measured in serum, urine, and dialysate samples of 32 chronic PD patients. Patients were thus screened for sarcopenia risk by the SARC-F tool, anthropometric measurements, and physical performance tests. *Results:* PD patients with a very high sarcopenia risk (SARC-F ≥ 2) had significantly lower serum (sMOTS-c) and higher dialysate (dMOTS-c) levels, suggesting an increased peritoneal clearance of this substance (d/s MOTS-c). sMOTS-c levels were directly correlated with muscle performance in physical tests, while an opposite relationship was found with dMOTS-c and d/sMOTS-c. ROC analyses demonstrated the diagnostic potential of MOTS-c, particularly in combination with physical and anthropometric assessments, to identify PD patients at very high risk of sarcopenia. *Conclusions:* Chronic PD may negatively affect MOTS-c balance, which, in turn, may contribute to enhanced sarcopenia risk. Larger studies are needed to confirm these observations and to validate the potential utility of this substance as a biomarker for improving sarcopenia risk stratification in PD patients.

## 1. Introduction

Sarcopenia, a condition characterized by a reduction in skeletal muscle mass along with reduced muscle strength and function, is pervasive in individuals with end-stage kidney disease (ESKD) on chronic dialysis treatment [1,2]. In peritoneal dialysis (PD), this disorder is less prevalent than in hemodialysis (HD) patients, but sarcopenic PD individuals exhibit an equally increased risk of falls, disability, cognitive decline, and mortality [3].

Uremic toxicity, malnutrition, volume overload, and protein leakage in peritoneal dialysate are the most important triggers of sarcopenia in PD. Still, several other factors related to the deranged metabolic and inflammatory state, or the uremic disequilibrium, may play a relevant role [4].

Mitochondrial dysfunction (MD) has recently gained increasing attention as a key hallmark of ESKD, which may contribute to uremic sarcopenia [5]. MD impairs bioenergetic cellular activity, particularly in tissues enriched in mitochondria like the heart and skeletal muscle, and could be revealed by altered homeostasis of mitochondrial-derived peptides (MDPs), small molecules encoded by the mitochondrial genome and endowed with important cellular functions [6].

The mitochondrial open reading frame of 12S rRNA-c (MOTS-c) is a small MDP known to regulate metabolism, enhance insulin sensitivity, and promote cellular adaptation to stress [7]. MOTS-c is primarily released by the skeletal muscle, acting as a positive inductor of muscle mass development [8]. Experimental MOTS-c administration reduces muscle wasting induced by the myostatin-mediated pathway, thus preventing sarcopenia [9], and physical exercise in humans enhances its biosynthesis, which, in turn, improves muscle function, metabolism, and trophism [10].

Of note, chronic inflammation impairs muscle MOTS-s expression in ESKD individuals [11], and altered circulating MOTS-c levels reflect cardiac dysfunction and predict worse CV outcomes among HD patients [12]. To date, however, the possible clinical significance of MOTS-c in individuals on chronic PD treatment, particularly concerning their sarcopenia risk, remains unexplored.

With this background in mind, we have conducted a pilot, observational study of PD patients to evaluate MOTS-c and the impact of dialysis treatment on its balance and body clearance. Afterward, we assessed the sarcopenia risk in the same individuals by a validated screening approach based on a simple and largely validated score tool, the SARC-F, and a series of clinical, anthropometric, and physical measurements. Finally, we tested possible correlations between sarcopenia risk measures and MOTS-c levels, and we investigated the potential usefulness of this peptide as a complementary biomarker to improve sarcopenia risk stratification.

## 2. Materials and Method

### 2.1. Patient Selection

From December 2023 to January 2024, 42 consecutive patients aged 18 years or older followed at the University Hospital of Ioannina and the University Hospital of Thessaloniki (Greece) who were on continuous ambulatory peritoneal dialysis (CAPD) or automated peritoneal dialysis (APD) for ESKD were screened for eligibility to take part in the study. Exclusion criteria were dialysis vintage < 6 months or switch from hemodialysis; active cancer; severe malnutrition or obesity; or any severe neurological, muscular, or skeletal disability.

### 2.2. Clinical Assessment and MOTS-c Measurement

Before beginning a scheduled outpatient visit, PD patients underwent a comprehensive clinical assessment, with a predetermined set of anthropometric, clinical, laboratory, and dialysis parameters being recorded. Biochemical data were measured using standard methods employed in the local clinical laboratory. MOTS-c was measured using an ELISA commercially available kit (My Biosource, San Diego, FL, USA), following the manufacturer’s instructions. Blood, urine, and peritoneal fluid samples were collected before physical testing (see below) and centrifuged at 1227× *g* for 15 min at 4 °C, with the aliquots being stored at −80 °C until thawed for batch analysis. The enzymatic reactions were quantified in an automatic microplate photometer and performed in the same laboratory (Clinical Pathology Lab, “Renato-Dulbecco” University Hospital, Catanzaro, Italy). MOTS-c levels were expressed as ng per mL. Additionally, the renal (u/sMOTS-c) and peritoneal (d/s MOTS-c) clearances of this substance were computed by the following formula:

dialysate [urine] MOTS-c (ng/mL)/serum MOTS-c (ng/mL) × 24-h dialysate [urine] volume (mL/day) × 1/1440 (day/min) × (1.73 m^2^ BSA)

### 2.3. Sarcopenia Risk Screening

Patients’ screening for sarcopenia risk was first made by the SARC-F questionnaire, a simple and largely validated tool [13] that assesses five key domains: strength, assistance with walking, rising from a chair, climbing stairs, and falls. Each item is scored on a scale from 0 to 2, where 0 indicates no difficulty, 1 indicates some difficulty, and 2 indicates significant difficulty or inability to perform the task. The total score ranges from 0 to 10, with higher scores indicating a greater risk of sarcopenia. After completing the SARC-F, patients underwent anthropometric analyses, and measurement of skinfold thickness at the tricep (in women), pectoral (in men), abdominal/supra-iliac, and thigh levels by a standard plicometer (Motivaly, Istanbul, Turkey). Lean and fat mass and body fat percentage were also estimated by the Jackson/Pollock and the Siri equations [14,15]. Further, upper body muscle strength was evaluated by a standard handgrip test using an electronic hand dynamometer (Kuptone, Shenzhen, China). The test was performed thrice on the same hand with a short rest period between trials. Force measurement was recorded in kilograms (kg), and the average of the three trials was used for analysis. Additionally, patients underwent a 30-s Chair Stand Test (30SCT) to assess lower body strength. During the test, the participants sat in the middle of a standard chair with their feet flat on the floor and arms crossed over their chest. Patients were instructed to stand up fully and sit back down as many times as possible within 30 s, without using their arms for support. The absolute number of completed stands within the allotted time was recorded and used for analysis.

### 2.4. Statistical Analysis

The statistical analysis was performed using the SPSS package (version 24.0.0.0; IBM corporation, Armonk, NY, USA), the MedCalc Statistical Software (version 14.8.1; MedCalc Software bvba, Ostend, Belgium), and the GraphPad Prism software (version 9.0.0; GraphPad Software LLC, La Jolla, CA, USA).

Differences between groups were assessed by the unpaired *t*-test for normally distributed values, the Mann–Whitney U test for non-parametric values, and the chi-square followed by a Fisher’s exact test for frequency distributions. Univariate correlations between sarcopenia risk measures and MOTS-c were assessed by the Pearson (R) coefficient. Before testing correlations, all skewed variables were log-transformed to approximate normal distribution.

Receiver Operating Characteristic (ROC) analyses were employed to assess the individual diagnostic capacity of MOTS-c and the various sarcopenia risk measures (anthropometrics, skinfold, and physical tests) in identifying patients with a higher SARC-F score, computing the area under the curve (AUC), and the best discriminatory cut-off value (Youden index). In addition, changes in the ROC-AUC, the explained variability (Nagelkerke R^2^), and the model calibration (Hosmer-Lemeshow X^2^) were assessed after incorporating discriminant MOTS-c measures into a combined diagnostic panel encompassing all the discriminant sarcopenia risk variables in individual ROC analyses.

All of the results were considered statistically significant for *p* values ≤ 0.05.

## 3. Results

### 3.1. Main Characteristics of the Study Cohort

The final study cohort consisted of 32 chronic PD patients. The main reasons for study exclusion were severe disability preventing physical testing (*n* = 8) and unwillingness to participate in the study (*n* = 2).

The mean age was 60.7 ± 1.2, and most individuals were male (62.5%). The prevalence of diabetes was 43.8%, and 37.5% of patients had prior CV disease. Chronic glomerulonephritis was the most frequent cause of ESKD requiring chronic dialysis (31.2%). The median dialysis vintage was 29 months [IQR 15–55]. Few patients were on Continuous Ambulatory Peritoneal Dialysis (CAPD; 37.5%), with the remaining being on nocturnal automated peritoneal dialysis (APD). The mean number of daily wells was 3.8 ± 1.2, with a median daily ultrafiltration of 700 [IQR 412.5–1075] mL/day. Patients displayed, on average, a more than satisfactory renal/dialysis adequacy (weekly Kt/V 2.73 ± 1.1) and had a median urine output of 1100 [500–1500] mL/day. In the latest peritoneal equilibration test (PET), most patients were slow to average transporters (43.7%), while the remaining were average to fast (37.5%) or fast (18.7%) transporters. The main lab exams were, on average, within the acceptable range for this population setting. Table 1 summarizes the main clinical, laboratory, and dialysis parameters of the study population.

### 3.2. MOTS-c in the Study Population

**Figure 1:**
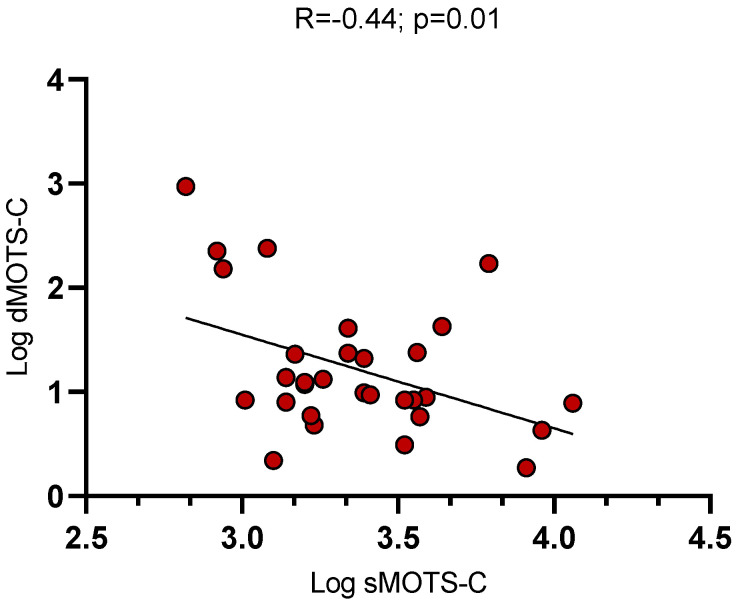
Linear correlations between (log-transformed) serum and dialysate MOTS-c levels.

On average, median MOTS-c levels in serum, urine, and dialysate samples were 28.7 [23.2–35.4], 1.39 [0.96–3.07], and 2.8 [2.3–4.2] ng/mL, respectively. An inverse, linear correlation was observed between sMOTS-c and dMOTS-c values (R = −0.44; *p* = 0.01; Figure 1), while no significant relationships were found between sMOTS-c and uMOTS-c (*p* = 0.32).

The estimated average renal (u/sMOTS-c) and peritoneal (d/sMOTS-c) MOTS-c clearances were 0.06 [0.03–0.10] and 0.08 [0.03–0.16] mL/min, respectively.

**Figure 2:**
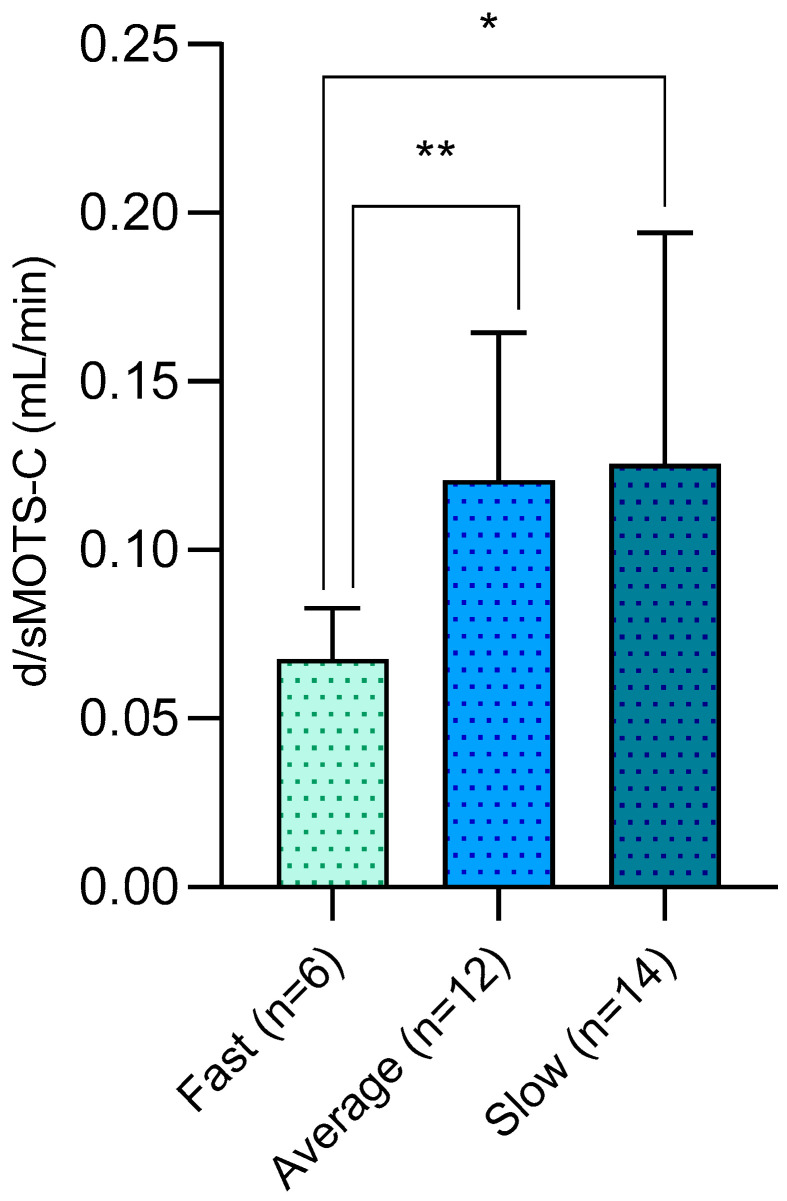
Difference in peritoneal MOTS-c clearance (d/sMOTS-c) among PD patients categorized for membrane transport performance at PET (peritoneal equilibration test). * *p* = 0.006; ** *p* = 0.03.

Considering the peritoneal membrane transport characteristics (in the PET test), d/sMOTS-c was significantly higher in “average” and even more in “slow” transporters compared with “fast” transporters (*p* = 0.03 and *p* = 0.006, respectively; Figure 2). On top of this, a barely significant difference in the d/sMOTS-c clearance (*p* = 0.09) emerged between patients on CAPD or APD.

### 3.3. Sarcopenia Risk in the Study Population

**Figure 3:**
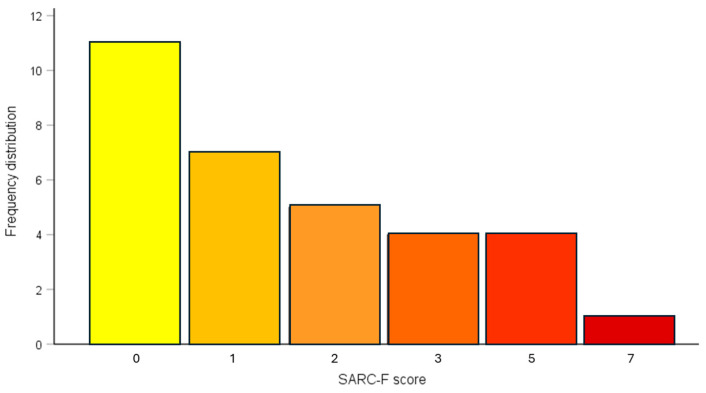
Frequency distribution of SARC-F scores in the study population.

The median SARC-F score in the study population was 1.75 [IQR range 1–3]. Visually, the overall frequency distribution was asymmetrically negative (Figure 3). Only 5/32 (15.6%) PD patients reported a SARC-F score ≥ 4, the cut-off value for high sarcopenia risk currently recommended in the general population [16], while 14/32 (40%) patients recorded a SARC-F score ≥ 2, a threshold that may reflect better sarcopenia risk in more frail populations [17,18,19].

In physical tests, handgrip strength was on average 23 ± 8.1 kg, while the average number of sit-to-stand repetitions in the 30SCT was 10.4 ± 3. At skinfold measurements, the supra-iliac and thigh thickness were 33.6 ± 11.3 and 38.4 ± 15.4 mm, respectively, while the mean triceps (in women) and the mean pectoral thickness (in men) were 40.9 ± 15.7 and 32.1 ± 10.5 mm, respectively.

The mean estimated lean mass was 49.1 ± 11.2 kg, while the relative percentage of body fat and the absolute fat mass were 35.2 ± 10.4% and 26.5 ± 8.8 kg, respectively.

Patients at very high sarcopenia risk (considering a SARC-F ≥ 2 cut-off) denoted poorer physical capacity in the handgrip test (*p* = 0.001), a barely significant reduced performance in the 30SCT (*p* = 0.08), as well as an increased supra-iliac skinfold (*p* = 0.03), higher body fat (*p* = 0.03), and lower lean mass (*p* = 0.01) when compared to others. Table 2 summarizes data on sarcopenia risk measures in the full study cohort and in subgroups of patients categorized as above reported.

### 3.4. Relationship Between MOTS-c and Sarcopenia Risk

**Figure 4:**
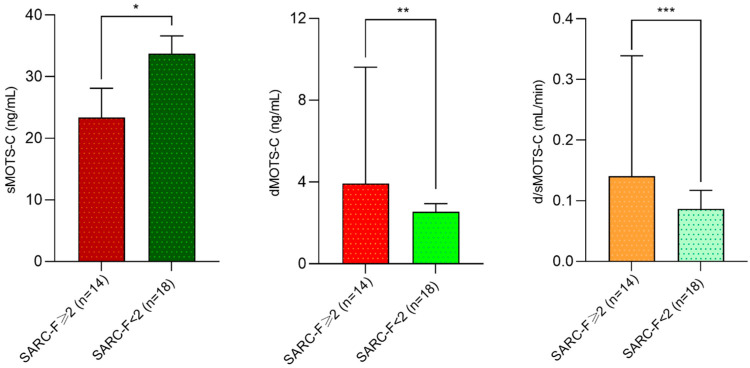
Differences in serum, dialysate, and peritoneal MOTS-C clearance between patients at very high risk of Sarcopenia (SARC-F ≥ 2) compared to others. * *p* = 0.003; ** *p* = 0.04; and *** *p* = 0.003.

PD patients with SARC-F score ≥ 2 displayed significantly reduced sMOTS-c (23.3 [19.8–28.1] vs. 33.7 [28.2–36.6] ng/mL; *p* = 0.003) and increased dMOTS-c (3.9 [2.3–1.6] vs. 2.5 [2.2–2.9] ng/mL *p* = 0.04) and d/sMOTS-c (0.15 [0.10–0.47] vs. 0.08 [0.06–0.11] mL/min; *p* = 0.003) compared to others (Figure 4). Conversely, no differences were observed for uMOTS levels and the renal MOTS-c (u/sMOTS-c) clearance (*p* > 0.05).

**Figure 5:**
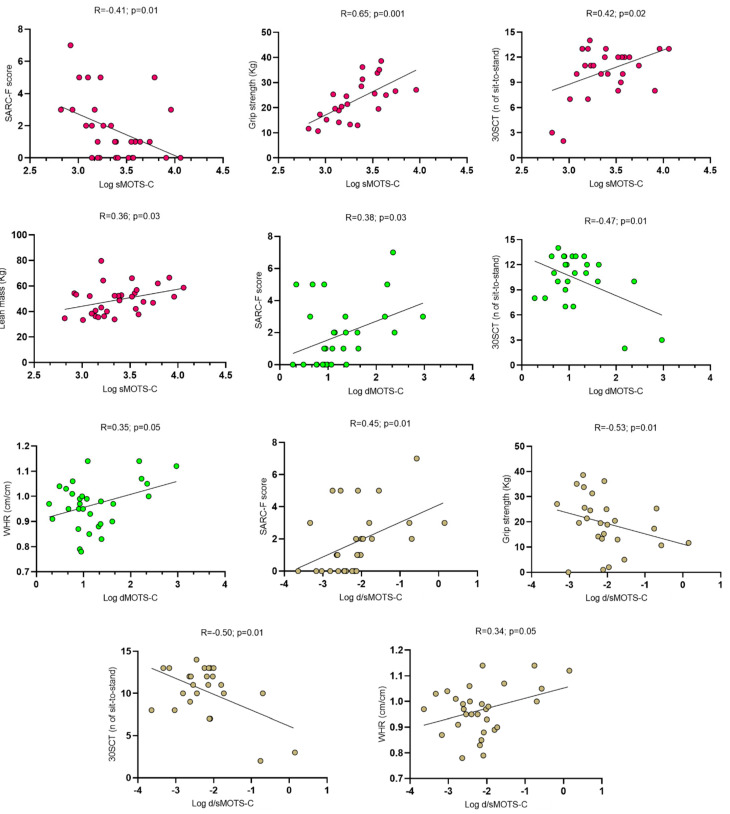
Significant relationships between sarcopenia risk correlates and MOTS-C.

Correlation analyses revealed direct relationships between sMOTS-c and, respectively, the physical performance in the handgrip test (R = 0.65; *p* = 0.001) and the 30SCT (R = 0.42; *p* = 0.02), and lean mass (R = 0.36; *p* = 0.03), while an inverse relationship was found with the absolute SARC-F score (R = −0.41; *p* = 0.01). dMOTS-c values were directly related to WHR (R = 0.35; *p* = 0.05) and the SARC-F score (R = 0.38; *p* = 0.03) and inversely related to performance in the 30SCT (R = −0.47; *p* = 0.01). Finally, direct correlations were evidenced between the d/sMOTS-c clearance and, respectively, the SARC-F score (R = 0.45; *p* = 0.01) and WHR (R = 0.34; *p* = 0.05), while inverse correlations were described with performance in the handgrip test (R = −0.53; *p* = 0.01) and the 30SCT (R = −0.50; *p* = 0.01). Figure 5 summarizes sarcopenia risk correlates of MOTS-c values.

### 3.5. Diagnostic Usefulness of MOTS-c for Sarcopenia Risk Stratification

In ROC analyses, grip strength, supra-iliac skinfold thickness, estimated lean mass, and body fat percentage all demonstrated a valuable diagnostic capacity in identifying PD patients at very high risk of sarcopenia (SARC-F ≥ 2) with correspondent AUCs ranging from 0.710 to 0.869 (*p* < 0.0001 to 0.02). The combination of these measures in a single risk panel led to a remarkable increase in diagnostic performance (AUC 0.938). Of note, this panel explained most of the risk variability in this cohort (71%), which resulted in it being well calibrated (X^2^ 7.02; *p* = 0.72).

**Figure 6:**
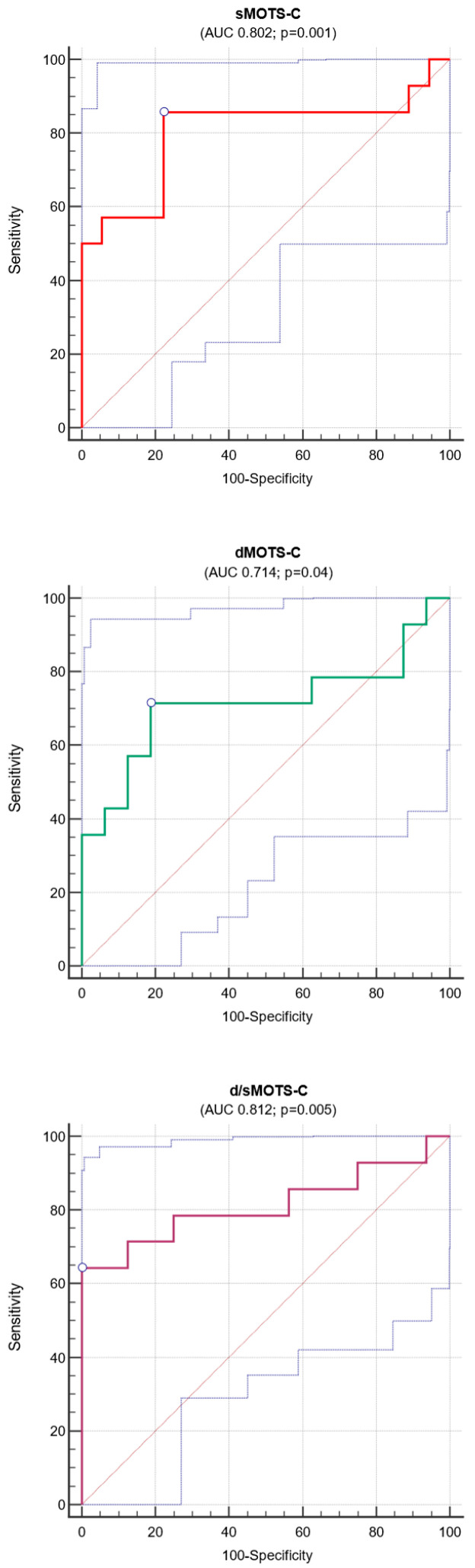
Diagnostic performance (area under the curve; AUC) of serum, dialysate, and peritoneal MOTS-C clearance for identifying PD patients at very high risk of sarcopenia (SARC-F ≥ 2).

Taken individually, sMOTS-c, dMOTS-c, and d/sMOTS-c were able to detect PD patients with SARC-F ≥ 2 in a significant manner (AUCs ranging from 0.714 to 0.812; *p* = 0.04–0.001. Figure 6), while uMOTS-c and u/sMOTS-c were not (AUCs ranging from 0.393 to 0.512; *p* > 0.05).

More importantly, when added to the combined panel, sMOTS-c, dMOTS-c, and d/sMOTS-c led to a gain in the overall diagnostic performance (AUCs 0.944 for sMOTS-c and 0.950 for both dMOTS-c and d/sMOTS-c), also keeping a good calibration (X^2^ ranging from 6.42 to 7.93). On top of this, d/sMOTS-c was also able to improve the explained risk variability slightly (74%). Table 3 summarizes data from diagnostic analyses.

## 4. Discussion

Various findings from our study deserve a focused discussion. First, we demonstrated that, in chronic PD patients, a significant quota of circulating MOTS-c undergoes body clearance by either the residual kidney function or the peritoneal dialysis treatment itself. To our knowledge, this is the first evidence in the literature providing insights into the clearance and overall balance of this substance in the complex ESKD-PD setting. Of note, the peritoneal MOTS-c clearance was barely affected by the PD technique (CAPD, APD) and, apparently, not influenced by the number/duration of daily wells or the dialysate composition. Given the pilot nature, our study could have been not sufficiently powered to capture measurable differences across different dialysis prescriptions; yet, a clear discrepancy in peritoneal MOTS-c clearance emerged after stratification for peritoneal membrane transport characteristics, with “fast transporters” in the PET test displaying a lower removal compared to others.

MOTS-c is generally considered a protective factor for CV health [20]. Notably, reduced circulating levels of MOTS-c have been observed in diabetic patients and have been associated with vascular dysfunction in obese children and individuals with recurrent angina [7]. Lower MOTS-c levels may predict impaired vascular recovery following myocardial infarction [21], while in chronic hemodialysis patients, unbalanced levels of this substance may predict worse CV outcomes, on top of traditional risk factors [12].

These observations underscore the importance of further investigating this substance in different clinical contexts. In patients undergoing chronic renal replacement therapy, including PD, targeted investigations are desirable to establish whether an increased MOTS-c removal with dialysis could have a detrimental effect, eventually driving worse clinical outcomes.

As mentioned earlier, despite alternative sites for its production having been postulated in ESKD [11,12], MOTS-c primarily remains a muscle-released factor that acts as a positive stimulator of trophism and function, eventually preventing sarcopenia [9,22,23]. Interestingly, in our PD cohort, circulating MOTS-c levels displayed inverse relationships with various sarcopenia risk measures, like the SARC-F score (the higher indicating a stronger sarcopenia risk) but also direct associations with muscle performance in physical tests and the estimated lean mass. By the same token, dialysate MOTS-c and peritoneal MOTS-c clearance values showed opposite relationships with muscle performance indicators and the SARC-F score. Previous studies of ESKD patients have demonstrated reduced MOTS-c expression at the muscle level, which, however, has not been investigated in relationship to muscle mass trophism or physical performance [11]. Given our observations, we may speculate that higher circulating MOTS-c levels in PD patients could in part reflect a better muscle status, particularly regarding the overall functionality, while higher peritoneal MOTS-c removal, also indicated by more increased MOTS-c levels in the dialysate fluid, may represent a negative finding, considering the beneficial effects of this substance on muscle health.

Unfortunately, the observational design of our investigation does not allow us to clarify the causality of this relation, which could only be explained by targeted experimental investigations. Moreover, the small sample size prevents the establishment of independent relationships by multivariate analyses, bearing in mind that MOTS-c levels could be affected by inflammation, malnutrition, and other co-morbidities [7,8,12]. Further, muscle biopsy for evaluating MOTS-c tissue expression has not been performed on the study participants, hampering the possibility of interpreting the clinical associations found.

As recommended, we screened patients for sarcopenia risk patients by a comprehensive and validated approach based on the SARC-F questionnaire and by collecting anthropometric and physical indicators of muscle status and function [24]. Nevertheless, the lack of sarcopenia confirmation by DXA, CT, or MRI scans to quantify muscle loss and the possible correlation of MOTS-c levels with these imaging measures remains another key limitation of our study, which merits to be specifically investigated by future research.

In the general population, a higher SARC-F score and a reduced muscle capacity in physical tests exhibit a very high concordance with “true” sarcopenia in imaging analyses [13], and a SARC-F ≥ 4 is commonly employed as the reference cut-off for identifying individuals at high risk for sarcopenia [16]. However, different studies have recently questioned the accuracy of this threshold, particularly in very old, frail, or multi-morbid subjects, in which a more inclusive cut-off (e.g., SARC-F ≥ 2) would best reflect the true severity of muscle impairment [17,18,19].

Indeed, in our study cohort, only 15.6% of PD patients reported a SARC-F score ≥ 4, while 14/32 (40%) patients recorded a SARC-F score ≥ 2. These latter evidenced significantly reduced muscle performance in physical tests and estimated lean mass; increased body fat percentage and supra iliac thickness; and, above all, lower circulating MOTS-c levels but higher dialysate levels and peritoneal clearance, compared to others. Of note, in ROC analyses either serum or dialysate levels and, particularly, peritoneal MOTS-c clearance values displayed a remarkable diagnostic capacity to identify PD patients at very high risk of sarcopenia (SARC-F ≥ 2). On top of this, the integration of MOTS-c values significantly increased the diagnostic performance of a combined panel including other functional and anthropometric sarcopenia risk measures. This observation is, in our view, of high importance as it may pave the way for larger studies examining the potential usefulness of MOTS-c as an easy, measurable, non-invasive tool for improving sarcopenia risk screening beyond traditional measures.

Considering the pilot and observational nature of our study, two more questions remain unsolved.

First, as alluded to before, it remains unclear whether MOTS-c holds a causal value on sarcopenia risk or just represents an epiphenomenon of the altered muscle status. Biological evidence of a causal relationship between lower blood MOTS-c levels and sarcopenia development may, for instance, set the stage for clinical trials testing recombinant MOTS-c for improving muscle mass and function [10,23,25]. Second, longitudinal analyses are needed to demonstrate whether an improvement in muscle conditions, e.g., after a tailored physical activity/dietary program, pairs with improved MOTS-c balance. Clarifying this point is critical to support the potential application of this substance as a biomarker for clinical monitoring.

## 5. Conclusions

We have demonstrated MOTS-c balance to be significantly affected by either peritoneal or renal clearance in patients undergoing chronic PD. In this population, MOTS-c may serve as a non-invasive biomarker for monitoring muscle status and may improve the risk assessment of sarcopenia. Future research in larger and more heterogeneous populations is advocated to generalize our findings and establish the potential usefulness of this substance for therapeutic purposes or as a biomarker for monitoring response to other structured therapeutic approaches for improving muscle health (e.g., physical exercise).

## Figures and Tables

**Table 1:** Main clinical parameters of the study population.

Age (yrs)	60.7 ± 1.2
Male gender (%)	62.5
Current smoking (%)	6.3
Diabetes (%)	43.8
History of CV disease (%)	37.5
Hypertension (%)	87.5
CKD etiology	
DKD (%)	6.25
Cardiorenal (%)	21.9
GNs (%)	31.2
Rare/ADPKD (%)	18.7
Other/Unknown (%)	21.9
Dialysis	
Dialysis vintage (mo.)	29 [15–55]
CAPD (%)	37.5
Daily wells (n.)	3.8 ± 1.2
PET (%fast/average/slow)	18.7/37.5/43.7
Kt/V	2.73 ± 1.1
UF (mL/day)	700 [412.5–1075]
Diuresis (mL/day)	1100 [500–1500]
Clinical/Lab	
Systolic BP (mmHg)	134.5 ± 20.3
Diastolic BP (mmHg)	79.3 ± 12
Serum creatinine (mg/dL)	6.1 ± 2.5
Urea (mg/dL)	116.4 ± 32.2
Glycemia (mg/dL)	96.8 ± 37
Albumin (g/dL)	3.8 ± 0.28
Serum sodium (mmol/L)	136.4 ± 4
Serum potassium (mmol/L)	4.45 ± 0.57
Serum calcium (mg/dL)	9.20 ± 0.71
Serum phosphate (mg/dL)	4.89 ± 1.23
Red blood cells (n ×10^6^)	4.14 ± 1.2
Haemoglobin (g/dL)	11.7 ± 1.1
Platelets (n × 10^3^)	255 ± 64.2
Total cholesterol (mg/dL)	171 ± 59.1
LDL cholesterol (mg/dL)	79 [62.2–118.2]
Triglycerides (mg/dL)	157.4 ± 66.3
iPTH (pg/mL)	205 ± 93.4
MOTS-c	
sMOTS-c (ng/mL)	28.7 [23.2–35.4]
uMOTS-c (ng/mL)	1.39 [0.96–3.07]
dMOTS-c (ng/mL)	2.8 [2.36–4.22]
u/sMOTS-c (mL/min)	0.06 [0.03–0.10]
d/sMOTS-c (mL/min)	0.08 [0.03–0.16]

Legend: CV, cardiovascular; DKD, diabetic kidney disease; GNs, glomerulonephritides, ADPKD, autosomal dominant polycystic kidney disease; CAPD, continuous ambulatory peritoneal dialysis; PET, peritoneal equilibration test; UF, ultrafiltration; BP, blood pressure; LDL, low-density lipoproteins; iPTH, intact parathormone; sMOTS-c, serum MOTS-c; uMOTS-c, urinary MOTS-c; u/sMOTS-c, renal clearance of MOTS-c; and d/s MOTS-c, dialysis clearance of MOTS-c.

**Table 2:** Measures of sarcopenia risk assessment in all patients and in subgroups of individuals categorized for a SARC-F cut-off of ≥2.

	All PD (*n* = 32)	SARC-F ≥ 2 (*n* = 14)	SARC-F < 2 (*n* = 18)	*p*
Grip strength (kg)	23 ± 8.1	17.8 ± 5.2	28.2 ± 7.1	0.001
30SCT (*n*. of sit-to-stand)	10.4 ± 3	9.1 ± 3.8	11.1 ± 2	0.08
BMI (Kg/m^2^)	26.5 ± 3.3	25.8 ± 3.3	27.1 ± 3.3	0.30
Waist-hip ratio (cm/cm)	0.97 ± 0.09	0.97 ± 0.1	0.97 ± 0.1	0.95
Triceps thickness (mm) *	40.9 ± 15.7	45 [13–51]	48 [41–52]	0.43
Pectoral thickness (mm) **	32.1 ± 10.5	33.1 ± 11.4	30.4 ± 9.3	0.60
Supra-iliac thickness (mm)	33.6 ± 11.3	38.4 ± 11	28.9 ± 10	0.03
Thigh thickness (mm)	38.4 ± 15.4	41.9 ± 13.9	35.7 ± 16	0.26
Body fat (%)	35.2 ± 10.4	39.5 ± 9.1	31.9 ± 10.2	0.03
Estimated lean mass (Kg)	49.1 ± 11.2	43.8 ± 9.8	53.3 ± 10.7	0.01
Estimated fat mass (Kg)	26.5 ± .8.8	27.8 ± 7.1	24.4 ± 9.9	0.46

Legend: 30SCT, 30-s chair test; BMI, body mass index. * in woman; ** in men.

**Table 3:** Individual and combined diagnostic profile (area under the curve-AUC) in detecting patients with SARC-F ≥ 2 of MOTS-c values and sarcopenia risk measures, which differed between study subgroups.

	AUC [95% CI]	*p*	Best Cut-Off	Sensitivity [95% CI]	Specificity [95% CI]
Single variables					
Grip strength	0.868 [0.668–0.970]	<0.0001	≤21.4	83.3 [51.6–97.9]	83.3 [51.6–97.9]
Supra-iliac thick	0.728 [0.543–0.870]	0.01	>35	71.4 [41.9–91.6]	72.2 [46.5–90.3]
Lean mass	0.740 [0.555–0.878]	0.01	≤40	57.1 [28.9–82.3]	94.4 [72.7–99.9]
Body fat	0.710 [0.524–0.856]	0.02	>40.6	57.1 [28.9–82.3]	83.3 [58.6–96.4]
sMOTS-c	0.802 [0.623–0.921]	0.001	≤28.2	85.7 [57.2–98.2]	77.8 [52.4–93.6]
dMOTS-c	0.714 [0.521–0.863]	0.04	>2.96	71.4 [34.8–93.3]	81.2 [61.5–99.8]
d/sMOTS-c	0.812 [0.629–0.931]	0.005	>0.132	64.2 [35.1–87.2]	100 [79.4–100]
	**AUC [95% CI]**	***p***	**R^2^**	**X^2^**	***p***
Combined panel					
Without MOTS-C	0.938 [0.759–0.995]	0.001	0.71	7.02	0.72
+sMOTS-C	0.944 [0.768–0.997]	0.002	0.71	7.93	0.63
+dMOTS-C	0.950 [0.765–0.998]	0.005	0.71	6.45	0.69
+d/sMOTS-C	0.950 [0.765–0.998]	0.003	0.74	6.42	0.69

Legend: sMOTS-c, serum MOTS-c; dMOTS-c, dialysate MOTS-c; d/sMOTS-c, dialysis clearance of MOTS-c.

## Data Availability

Raw data are available from the corresponding author upon reasonable request.
